# RAS Mutations as Prognostic Biomarkers in Digestive Cancers: From Binary Classification to Variant-Specific and Context-Dependent Risk Stratification

**DOI:** 10.7759/cureus.116374

**Published:** 2026-09-16

**Authors:** Lucien Grados, Rami Rhaiem, Koceila Amroun, Perrine Zimmermann, Sophie Deguelte, Elia Gigante, Olivier Bouche, Reza Kianmanesh

**Affiliations:** 1 Hepatogastroenterology and Digestive Oncology, CHU de Reims, University of Reims Champagne-Ardenne, Reims, FRA; 2 General, Digestive and Endocrine Surgery, CHU de Reims, University of Reims Champagne-Ardenne, Reims, FRA; 3 Medical Oncology, Robert Debré Hospital, CHU de Reims, University of Reims Champagne-Ardenne, Reims, FRA; 4 General, Digestive and Endocrine Surgery, Cabrol Hospital, CHU de Reims, University of Reims Champagne-Ardenne, Reims, FRA

**Keywords:** anti-kras, cholangiocarcinoma, colorectal cancer, ctdna, gastrointestinal cancers, kras, kras mutation, nras mutations, pancreatic ductal adenocarcinoma, prognosis

## Abstract

In digestive cancers, activating RAS mutations - primarily KRAS, less frequently NRAS - are among the most prevalent oncogenic drivers. Their clinical significance depends strongly on the tumor type, disease stage, treatment context, and specific RAS variant. In colorectal cancer (CRC), RAS alterations are well-established predictors of resistance to anti-EGFR therapy. However, KRAS G12C-mutant tumors represent a distinct subgroup with demonstrated sensitivity to KRAS G12C inhibitors. However, growing evidence supports the negative prognostic impact of RAS mutations on both primary CRC and colorectal cancer liver metastases (CRLM) in both post-resection and adjuvant therapies. In pancreatic ductal adenocarcinoma (PDAC), where KRAS alterations are nearly universal, prognostic evaluation has evolved from a binary mutant versus wild-type classification to a more refined approach based on mutation subtype (e.g., G12D/V vs. G12R) and KRAS mutant dosage, both of which influence clinical outcomes. In biliary tract cancers, KRAS mutations occur in a relevant subset and are consistently associated with poor survival. Liquid biopsy approaches increasingly enable noninvasive detection and longitudinal monitoring. In gastric cancer, RAS alterations are less frequent overall but may identify biologically aggressive subsets and may also be associated with inferior outcomes under HER2-targeted therapy in selected populations.

This review synthesizes contemporary data supporting RAS alterations as prognostic biomarkers across digestive cancers, highlights practical considerations for integrating variant-level RAS information into risk stratification and trial designs, and discusses how emerging RAS-directed therapies may reshape the prognostic significance of specific molecular subsets.

## Introduction and background

RAS proteins (KRAS/NRAS/HRAS) are small GTPases that transduce signals from receptor tyrosine kinases to downstream effectors, including the MAPK and PI3K pathways. They are involved in cell proliferation and differentiation. Oncogenic RAS mutations “lock” RAS in an active GTP-bound state, driving proliferation, invasion, and therapy resistance in cancer cells. In digestive oncology, KRAS is the dominant isoform, while NRAS contributes particularly to CRC, and HRAS remains rare. The prognostic impact of RAS alterations is not uniform and reflects, at least in part, the interplay between intrinsic tumor biology and treatment-driven selection effects (e.g., exclusion of anti-EGFR therapy in RAS-mutant colorectal cancer) [1,2]. Consequently, interpreting RAS as a prognostic biomarker requires explicit attention to the disease setting and therapy exposure.

## Review

Scope and literature selection

This narrative review focused on the prognostic significance of somatic RAS alterations in digestive malignancies, including colorectal cancer, pancreatic ductal adenocarcinoma, biliary tract cancers, and gastric or gastroesophageal adenocarcinoma. A PubMed search was performed using combinations of the terms “RAS”, “KRAS”, “NRAS”, “prognosis”, “survival”, “colorectal cancer”, “pancreatic cancer”, “pancreatic ductal adenocarcinoma”, “cholangiocarcinoma”, “biliary tract cancer”, “gastric cancer”, “gastroesophageal adenocarcinoma”, “ctDNA”, and “mutant dosage”. English-language human studies published up to August 2026 were considered. Priority was given to systematic reviews and meta-analyses, large prospective or retrospective cohorts, clinicogenomic studies, and studies specifically reporting variant-level or quantitative RAS-associated survival outcomes. Small case series, preclinical studies, studies without relevant survival outcomes, and reports focused exclusively on non-digestive malignancies were excluded from the prognostic evidence synthesis. Recent clinical studies of RAS-directed therapies were additionally considered when relevant to the evolving distinction between prognostic and predictive implications of RAS alterations.

Prognostic versus predictive: why RAS is often misclassified

A key conceptual pitfall is the conflation of predictive and prognostic effects. Predictive biomarkers can identify treatment sensitivity or resistance. In metastatic colorectal cancer, RAS mutations define resistance to anti-EGFR monoclonal antibodies (cetuximab and panitumumab), a finding consistently supported by randomized trial meta-analyses and central to treatment decision-making [1,3].

Prognostic biomarkers provide information on patient outcomes independent of treatment exposure. Beyond their established predictive role, RAS mutations are associated with poorer outcomes, although the strength and consistency of this association vary across disease settings and study populations [1]. In contemporary CRC cohorts shaped by biomarker-driven treatment allocation, survival differences between RAS-defined groups may arise as much from differential access to effective therapies as from intrinsic tumor biology, thereby introducing significant treatment-related confounding factors. This limitation is less pronounced in pancreatic ductal adenocarcinoma (PDAC), where the near-universal prevalence of KRAS alterations and the historical lack of KRAS-guided treatment selection have limited treatment-related confounding. However, this paradigm is now evolving with the development of variant-directed therapies. Accordingly, throughout this review, associations observed within treatment-defined cohorts are interpreted as prognostic or treatment-contextual unless a formal biomarker-by-treatment interaction demonstrates a predictive effect.

Localized colorectal cancer

Stage II-III Colon Cancer: Evidence for a True Prognostic Signal

In the adjuvant setting, where treatment selection is less dominated by RAS status than in metastatic disease, a systematic review and meta-analysis of stage II-III colon cancer found that KRAS mutations were associated with worse disease-free and overall survival, alongside BRAF mutation effects, with signals influenced by microsatellite instability adjustment [4]. More recent variant-level analyses have further suggested that the prognostic effect of KRAS may not be uniform across individual mutations. In two independent cohorts of patients with resected stage II-III CRC, the uncommon KRAS G12A variant was associated with early recurrence and inferior recurrence-free and overall survival, supporting additional prognostic heterogeneity within KRAS-mutant disease [5].

Practical implications: For resected stage II-III disease, KRAS status can contribute to risk stratification but should be interpreted alongside other dominant factors (T/N stage, MSI, lymphovascular invasion, and emerging circulating tumor DNA-based minimal residual disease).

Metastatic CRC: population-level prognosis and variant-level nuance

In a large Nordic real-world pooled analysis of metastatic CRC, KRAS mutations were observed in approximately half of the molecularly classified tumors, NRAS mutations in a smaller subset, and survival differed across molecular groups: RAS and BRAF wild-type had the longest overall survival, KRAS- and NRAS-mutant groups were intermediate, and BRAF V600E-mutant tumors had the poorest survival [2]. Importantly, the analysis suggested limited clinically relevant differences between common RAS mutations, with only selected variant-specific signals (e.g., KRAS G12S) emerging in some comparisons.

In parallel, institutional cohort analyses have reported that NRAS-mutant CRC represents a distinct molecular subset with adverse outcomes compared to RAS wild-type CRC [6].

Interpretation: The marked survival disadvantage associated with BRAF V600E underscores that not all MAPK pathway alterations are prognostically equivalent. In contrast, KRAS- and NRAS-mutant tumors generally exhibit an intermediate prognostic phenotype. However, treatment-related confounding should be acknowledged because patients harboring RAS mutations are not eligible for anti-EGFR therapy, which may partly contribute to observed survival differences.

Colorectal Liver Metastases (CRLM): RAS and Outcomes After Resection

Among patients undergoing CRLM resection, multiple studies have shown that KRAS mutations are associated with poorer long-term outcomes, a finding supported by meta-analytic evidence demonstrating inferior survival after hepatectomy [7].

Beyond a binary mutant-wild-type classification, variant-level analyses indicate that specific KRAS codon 12 mutations may further refine prognostic stratification, underscoring the clinical relevance of RAS subtype [8].

Clinical takeaway: KRAS status, and potentially codon- or variant-level information, is increasingly incorporated into multidisciplinary decision-making regarding surgical candidacy, recurrence risk, and surveillance strategies, although its integration into standardized risk models remains inconsistent.

Pancreatic ductal adenocarcinoma

From “KRAS Mutant” to “Which KRAS Mutation?”: Variant-Specific Prognosis

PDAC is characterized by a very high prevalence of KRAS alterations. Therefore, prognostic refinement has shifted toward mutation-specific analyses that integrate both KRAS allele identity and underlying tumor biology.

In a large clinicogenomic cohort of metastatic PDAC, clinical outcomes differed according to KRAS G12 subtype, with G12R associated with more favorable survival, whereas G12D and G12V were linked to higher risks of progression and death. These associations are further modulated by the treatment regimen (e.g., FOLFIRINOX versus gemcitabine-based therapy) [9].

Similarly, in a large surgical cohort with integrated molecular analyses, KRAS G12R tumors were enriched in earlier-stage disease and were associated with reduced distant recurrence and improved survival compared with KRAS G12D tumors, along with distinct transcriptomic and functional profiles supporting mutation-specific biology [10]. These observations are supported by additional large cohorts. In an institutional cohort with external validation, KRAS G12D and Q61 alterations were associated with shorter survival, whereas G12R showed a comparatively favorable clinical profile. However, not all series have demonstrated an independent survival difference across KRAS subtypes, suggesting that disease stage, co-mutations, treatment exposure, and population characteristics may modulate their prognostic effect [11,12]. Conversely, recent real-world data in patients receiving first-line NALIRIFOX identified KRAS G12D and G12V as adverse prognostic factors, further supporting the potential clinical relevance of variant-level stratification [13].

Implication: These findings indicate that KRAS allele identity is a clinically relevant determinant of disease behavior in PDAC, with G12R defining a distinct subgroup characterized by less aggressive metastatic patterns and more favorable outcomes, thereby supporting mutation-specific stratification in clinical trials and prognostic models. Importantly, the prognostic implications of KRAS subtypes in PDAC may evolve with the emergence of mutation-directed therapies [14,15]. Pan-RAS inhibitors such as daraxonrasib (RMC-6236) and KRAS G12D-selective inhibitors currently under clinical investigation may progressively modify the natural history of specific molecular subsets and further strengthen the rationale for mutation-oriented stratification in future studies.

KRAS Mutant Dosage: An Additional Layer of Prognostic Stratification

Beyond mutation subtypes, KRAS mutant dosage, reflecting allelic imbalance, copy number alterations, and clonal architecture, has emerged as an additional prognostic biomarker in pancreatic cancer. In a large clinicogenomic analysis, a higher KRAS mutant dosage was associated with adverse clinical outcomes and distinct biological features, supporting its role as an independent prognostic factor [16].

Mechanistically, KRAS dosage may capture tumor evolutionary dynamics that are not reflected by single hotspot mutations alone, including clonal expansion and genomic amplification. This may partly explain the heterogeneity in clinical outcomes observed among patients harboring the same KRAS amino acid substitutions.

Biliary tract cancers and cholangiocarcinoma

KRAS mutations occur in a clinically significant subset of biliary tract cancers and are increasingly supported as adverse prognostic biomarkers. Recent large genomic and clinicogenomic studies have demonstrated substantial heterogeneity according to primary tumor site and KRAS allele. In particular, G12 variants have been associated with poorer survival, although the magnitude of this effect varies across intrahepatic and extrahepatic cholangiocarcinoma and gallbladder cancer. In resected intrahepatic cholangiocarcinoma, KRAS G12 variants were independently associated with inferior overall and disease-free survival, with G12V showing the strongest adverse prognostic signal [17]. Large contemporary cohorts, including analyses of several thousand patients, have further confirmed that KRAS-mutant BTC represents a clinically distinct subgroup with adverse survival outcomes and variant-specific heterogeneity, a finding also supported by meta-analytic evidence in resected cholangiocarcinoma [18-21]. In a large analysis integrating tissue-based genomics and circulating tumor DNA (ctDNA), KRAS driver mutations were identified in a meaningful proportion of cases and were associated with inferior overall and recurrence-free survival [22]. The study also demonstrated the feasibility of detecting KRAS G12/G13 variants in plasma, with a higher mutant allele frequency (MAF) correlating with worse outcomes, particularly when combined with elevated CA19-9 levels.

Clinical implications: In biliary tract cancers, KRAS status may serve as a prognostic biomarker, while ctDNA-based detection may offer a complementary tool for longitudinal molecular monitoring, particularly when tissue availability is limited [22].

Gastric cancer and gastroesophageal adenocarcinoma

RAS mutations are less frequent in gastric cancer and gastroesophageal adenocarcinomas than in CRC or PDAC; however, available data suggest that they may define biologically and clinically relevant subgroups. In HER2-positive advanced gastric cancer treated with trastuzumab-based chemotherapy, KRAS mutations have been associated with lower response rates and shorter progression-free and overall survival. These findings suggest that KRAS mutations may identify a subgroup with poorer outcomes during HER2-targeted therapy; however, in the absence of a formal biomarker-by-treatment interaction, this association should not be interpreted as demonstrating a predictive effect [23]. Similarly, in metastatic or recurrent gastric cancer treated with first-line S-1 plus cisplatin, KRAS mutation status (and MET amplification) has been associated with prognostic differences in reported cohorts, supporting the broader principle that MAPK pathway activation may influence disease behavior and treatment response [24].

Interpretation: Overall, the prognostic evidence for RAS alterations in gastric and gastroesophageal adenocarcinoma remains limited and inconsistent. Some cohorts have associated KRAS alterations with inferior outcomes in selected settings, whereas others have failed to demonstrate an independent prognostic effect [23-26]. This heterogeneity, together with the relatively low prevalence of KRAS mutations compared with CRC and PDAC, currently limits the clinical utility of RAS status as a standalone prognostic biomarker in gastric cancer.

Implementation considerations and pitfalls

Several methodological and clinical considerations should be acknowledged when interpreting the prognostic value of RAS alterations in digestive cancers.

Assay heterogeneity remains a critical limitation. Differences in sequencing depth, targeted panels versus next-generation sequencing (NGS), and ctDNA assay design may influence the detection of RAS alterations, their apparent prevalence, and clonal fraction, thereby affecting prognostic modeling.

Treatment confounding is a major concern, particularly in colorectal cancer. Because RAS status determines eligibility for anti-EGFR therapies, the observed survival differences between RAS-defined groups may reflect differential access to effective treatments rather than intrinsic tumor biology. Meta-analytic data help disentangle predictive and prognostic contributions.

Variant-level and quantitative features represent the next step in the refinement of prognostic tools. In PDAC, emerging data support both KRAS allele specificity and KRAS mutant dosage as clinically relevant prognostic layers beyond a binary mutation call.

Finally, tumor type specificity is essential. The prognostic implications of a given KRAS variant are not uniform across tumor types, reflecting differences in cellular origin, co-mutations, and therapeutic context.

Future directions

Future research should focus on developing multidimensional prognostic frameworks combining RAS variant information with quantitative biomarkers, ctDNA minimal residual disease measurements, clinicopathological variables, and co-mutation profiles. Such integrated models could potentially improve prognostic stratification compared with single biomarkers alone, but this hypothesis requires formal validation. Mutation-specific trial stratification should be encouraged. In PDAC, consistent clinical and biological differences between KRAS subtypes, particularly G12R and G12D/V, support the development of dedicated stratification strategies in prospective studies. Prospective validation across underexplored tumor types is necessary. In biliary tract and gastric cancers, current evidence is heterogeneous and largely derived from retrospective cohorts, underscoring the need for standardized genomic profiling and prospective outcome assessments.

The present review complements previous disease-specific analyses by providing a cross-tumour synthesis of the prognostic significance of RAS alterations. Formica et al. established the adverse prognostic impact of KRAS mutations in stage II-III colon cancer, whereas McIntyre et al. demonstrated distinct clinical and biological outcomes across KRAS variants in resected PDAC [4,10]. More recently, Aslani et al. confirmed in a systematic review and meta-analysis that the prognostic impact of individual KRAS variants in PDAC varies according to disease stage and treatment context [27]. By integrating evidence across colorectal, pancreatic, biliary tract, and gastric cancers, our review highlights that the prognostic significance of RAS alterations depends not only on mutation status or variant identity, but also on tumour type, disease stage, quantitative allelic features, and treatment context (Table 1).

**Table 1:** Representative clinical studies evaluating the prognostic significance of RAS alterations across digestive cancers. Effect estimates are presented with 95% confidence intervals whenever available. Adjusted estimates are preferentially reported; when unavailable, unadjusted estimates are explicitly identified. NR indicates information not reported in the original publication. Associations observed within a specific treatment cohort are considered prognostic or treatment-contextual unless a formal biomarker-by-treatment interaction demonstrates a predictive effect. Abbreviations: aHR, adjusted hazard ratio; BTC, biliary tract cancer; CCA, cholangiocarcinoma; cfDNA, cell-free DNA; CGP, comprehensive genomic profiling; CI, confidence interval; CRC, colorectal cancer; CRLM, colorectal liver metastases; CT, chemotherapy; DFS, disease-free survival; EHC, extrahepatic cholangiocarcinoma; FFPE, formalin-fixed paraffin-embedded; FISH, fluorescence in situ hybridization; GB, gallbladder cancer; GC, gastric cancer; HER2, human epidermal growth factor receptor 2; HR, hazard ratio; IHC, intrahepatic cholangiocarcinoma; MAF, mutant allele frequency; mCRC, metastatic colorectal cancer; MMR, mismatch repair; MSI, microsatellite instability; NGS, next-generation sequencing; OS, overall survival; PDAC, pancreatic ductal adenocarcinoma; PFS, progression-free survival; PS, performance status; RFS, recurrence-free survival; TTNT, time to next treatment; WES, whole-exome sequencing; WGD, whole-genome doubling; WT, wild type.

Study	Design; N; stage/setting	Assay source	Comparator	Endpoint	Effect estimate (95% CI)	Follow-up	Adjustment variables	Main conclusion
Osterlund et al., 2025 [2]	Population-based cohort; N=2,118; mCRC	Tumour; routine testing	KRAS-mut / NRAS-mut / BRAF V600E vs RAS/BRAF WT	OS	NR	NR	NR	Median OS: WT 28.3, KRAS 21.4, NRAS 26.3, BRAF 9.2 mo
Formica et al., 2022 [4]	Meta-analysis; N=10,893; stage II–III CRC	Tumour; assays varied	KRAS-mut vs WT	DFS; OS	DFS HR 1.36 (1.15–1.61); OS HR 1.27 (1.03–1.55)	NR	Trial-level multivariable estimates; MSI-stratified	Adverse DFS/OS; precise pooled estimates
Momma et al., 2025 [5]	Two retrospective cohorts; N=299 + 178; resected stage II–III CRC	Tumour; molecular testing/WES	G12A vs WT	RFS; OS	Institutional: RFS aHR 5.23 (1.50–14.11), OS 10.64 (2.39–34.00); external: RFS 5.13 (1.15–16.44), OS 3.83 (1.08–10.61)	NR	Age, sex, site, histology, stage, adjuvant CT, MMR/MSI	Marked adverse association; wide CIs
Brudvik et al., 2015 [7]	Meta-analysis; N=1,809; resectable CRLM	Tumour; assays varied	KRAS-mut vs WT	OS; RFS	OS HR 2.24 (1.76–2.85); RFS HR 1.89 (1.54–2.32)	NR	Pooled multivariable estimates; covariates varied	~2-fold mortality and ~89% higher recurrence hazards
Margonis et al., 2015 [8]	Retrospective cohort; N=331; resected CRLM	Tumour; KRAS codon 12/13	Specific variants vs WT	OS	G12V HR 1.78 (1.00–3.17); G12S HR 3.33 (1.22–9.10), unadjusted	Median 27.4 mo	Unadjusted variant-specific estimates	Higher mortality with G12V/G12S; imprecise for G12S
Aslani et al., 2026 [27]	Meta-analysis; 15 studies; early-/late-stage PDAC	Tumour; assays varied	G12D/G12R/G12V vs study-specific reference	OS	G12D HR 1.64 (1.28–1.99); +CT 1.29 (1.18–1.39); G12R +CT 0.74 (0.56–0.99); early G12V 0.67 (0.52–0.86)	Varied	Study-level estimates; stage/treatment subgroups	Variant effects were stage- and treatment-context dependent
McIntyre et al., 2024 [10]	Retrospective cohort; N=1,360; resected PDAC	Tumour genomic profiling; RNA/spatial subsets	G12R/G12V vs G12D	OS	G12R aHR 0.77 (0.60–0.99); G12V 0.71 (0.58–0.88)	NR	KRAS variant, tumour-suppressor alterations; stratified by source	G12R/G12V associated with ~23%/~29% lower mortality hazards
Yousef et al., 2024 [11]	Retrospective cohort + external validation; N=803 + 408; all-stage PDAC	Tumour genomic testing	G12D/Q61/G12R vs WT	OS	G12D HR 1.70 (1.30–2.30); Q61 1.90 (1.20–3.00); G12R 1.00 (0.71–1.50)	NR	NR for reported variant estimates	Adverse G12D/Q61; no clear excess risk with G12R
Norton et al., 2025 [9]	Clinicogenomic cohort; N=2,433; metastatic PDAC	Tumour sequencing + longitudinal data	G12D/G12V vs WT	OS; TTNT	OS: G12D aHR 1.29 (1.15–1.45), G12V 1.23 (1.09–1.39); TTNT: 1.15 (1.04–1.29), 1.16 (1.04–1.30)	NR	Multivariable Cox; KRAS, co-mutations, treatment variables	G12D/G12V associated with ~20–30% higher mortality hazards
Cho et al., 2025 [12]	Multicenter retrospective cohort; N=269; advanced PDAC	Tumour molecular profiling	KRAS variants; treatment-specific comparisons	OS; PFS	NR for robust variant-specific adjusted OS estimate	NR	KRAS variant, CT regimen, variant×treatment interaction	No robust variant-specific OS effect; predictive signals exploratory
Reichinger et al., 2025 [13]	Multicenter real-world cohort; N=80; first-line NALIRIFOX mPDAC	Tumour molecular profiling	G12D/G12V vs other KRAS/WT	OS	NR	NR	NR for KRAS-specific prognostic comparison	G12D/G12V associated with poorer OS; treatment-contextual, not predictive
Varghese et al., 2025 [16]	Clinicogenomic cohort; N=2,336; all-stage PDAC	Tumour; MSK-IMPACT NGS	Mutant-allele dosage gain vs one mutant copy	OS	Overall non-WGD aHR 1.70 (1.40–2.00); resectable 2.16 (1.10–4.30); metastatic 1.63 (1.30–2.10)	Median NR	Sex, age, ancestry, diagnosis-to-sampling time	Dosage gain associated with ~70% higher mortality hazard
Zhou et al., 2022 [17]	Retrospective cohort; N=1,024; resected IHC	Tumour; KRAS variant analysis	G12 vs WT; G12V analysis	OS; DFS	G12: OS aHR 1.69 (1.31–2.18), DFS 1.47 (1.16–1.88); G12V: OS 3.05 (1.94–4.79), DFS 1.79 (1.13–2.85)	NR	Multivariable; clinicopathological covariates	Adverse G12 effect; largest mortality estimate for G12V
Gelfer et al., 2024 [20]	Clinicogenomic cohort; N=985; BTC	Tumour genomic profiling	G12 vs WT	OS	IHC aHR 1.98 (1.41–2.79); EHC 0.74 (0.47–1.16); GB 0.64 (0.20–2.07)	NR	Age, stage, multifocality, curative surgery	Adverse association confined to IHC
Moffat et al., 2024 [19]	Multicenter clinicogenomic cohort; N=7,457	Tumour NGS	G12D/G12V/Q61H/other variants	OS	No aHR; KM/log-rank only	NR	No multivariable survival adjustment	Allele-specific survival differences; interpretation limited by no adjustment
Thongyoo et al., 2025 [22]	Translational cohort; N=937; CCA; cfDNA N=101	Tumour sequencing; plasma cfDNA	KRAS-mut vs WT; higher vs lower MAF	OS; RFS	NR	NR	NR	KRAS mutation/higher MAF associated with poorer OS/RFS
Iida et al., 2025 [18]	Nationwide cohort; N=7,773; BTC	Tumour CGP	Specific variants vs WT	OS	IHC: G12D aHR 1.83 (1.52–2.20), G12V 1.37 (1.05–1.78), Q61H 2.03 (1.41–2.91); EHC: G12D 1.39 (1.12–1.73), G12V 1.58 (1.25–2.00)	NR	Age, PS, first-line treatment, curative surgery	Variant effects differed by anatomical subtype; largest for IHC Q61H
Shimozaki et al., 2023 [23]	Retrospective cohort; N=77; HER2+ advanced GC	Tumour molecular testing	KRAS-mut (n=6) vs WT (n=71)	Response; PFS; OS	NR	NR	NR	Poorer outcomes with KRAS mutation; predictive value not established
Matsusaka et al., 2016 [24]	Retrospective cohort; N=150; metastatic/recurrent GC	Tumour molecular analysis	KRAS-mut vs WT	PFS; OS	NR	NR	NR for KRAS-specific estimates	Poorer PFS/OS; precision limited by very small mutant subgroup
Takahashi et al., 2014 [25]	Retrospective cohort; N=167; advanced GC	FFPE/frozen tumour; sequencing	NRAS-mut/KRAS-mut vs WT	OS	NRAS aHR 5.61 (1.64–19.20); no clear adjusted KRAS effect	NR	Multivariable survival model	Large adverse NRAS estimate but wide CI; no independent KRAS effect
Rehkaemper et al., 2020 [26]	Tissue-based retrospective cohort; N=582; GC	Tumour; FISH/IHC	KRAS-amplified vs non-amplified	OS	NR	Median 26 mo among survivors	No aHR for principal comparison	No overall OS difference; selected resected subgroup 16 vs 35 mo

The prognostic significance of KRAS alterations in PDAC may also evolve rapidly with the emergence of effective RAS-directed therapies. The phase III RASolute 302 trial recently demonstrated a marked overall survival advantage of the pan-RAS(ON) inhibitor daraxonrasib over standard second-line chemotherapy in metastatic PDAC, establishing RAS inhibition as a new therapeutic paradigm in this disease [15]. In parallel, the ongoing phase III RASolute 303 study is evaluating daraxonrasib alone or in combination with gemcitabine and nab-paclitaxel as first-line therapy for metastatic PDAC. Selective KRAS G12D inhibition is also advancing rapidly. The phase III RASolute 305 trial is evaluating first-line chemotherapy with or without zoldonrasib (RMC-9805) in metastatic KRAS G12D-mutated PDAC. Beyond genomic RAS alterations, emerging regulatory biomarkers such as circular RNAs may provide complementary information on tumour biology and prognosis. Their stability and regulatory functions, including interactions with microRNAs and signalling networks, make them attractive candidates for future multimodal biomarker models, although their incremental prognostic value in RAS-driven digestive cancers remains to be established.

Consequently, prognostic differences previously attributed to intrinsic biological properties of KRAS variants may increasingly reflect differential access to mutation-directed therapies, warranting periodic reassessment of KRAS-based prognostic models. At this time, this integrated risk framework remains conceptual and has not undergone formal statistical development or validation; it should therefore not be considered ready for clinical implementation. Future studies should prospectively evaluate its discrimination and calibration, assess proportional-hazards assumptions and competing risks where appropriate, perform internal and external validation, and determine whether RAS-based variables provide incremental prognostic value beyond established clinicopathological factors.

## Conclusions

Across digestive cancers, RAS alterations represent more than treatment-selection biomarkers; they also carry meaningful prognostic information, but in a context-dependent and increasingly variant-specific manner. In CRC, KRAS/NRAS mutations are associated with intermediate survival in metastatic settings and adverse outcomes in selected settings, such as stage II-III disease and CRLM resection. In PDAC, the most clinically relevant prognostic insights are derived from KRAS allele identity (e.g., G12R vs. G12D) and KRAS mutant dosage, which reflect distinct biological programs and disease trajectories. In biliary tract cancers, recent large cohorts increasingly support an adverse and potentially variant-specific prognostic effect of KRAS alterations, whereas evidence in gastric and gastroesophageal cancers remains more limited and heterogeneous. Future efforts should prioritize prospective validation and determine whether integrating RAS variant-level and quantitative features into multiparameter risk models provides incremental prognostic value beyond established clinicopathological factors. Importantly, the recent demonstration of a survival benefit with daraxonrasib in previously treated metastatic PDAC suggests that the prognostic significance of KRAS alterations may become increasingly treatment-dependent, reinforcing the need for continuous updating of RAS-based prognostic frameworks.
